# Trapping evidence for the thermal cyclization of di-(*o*-acetylphenyl)acetylene to 3,3'-dimethyl-1,1'-biisobenzofuran

**DOI:** 10.1186/1860-5397-1-18

**Published:** 2005-12-09

**Authors:** Charles P Casey, Neil A Strotman, Ilia A Guzei

**Affiliations:** 1 University of Wisconsin-Madison, Madison, WI 53706

## Abstract

The reaction of di-(*o*-acetylphenyl)acetylene (**1**) with excess dimethyl acetylenedicarboxylate (DMAD) produced bis-DMAD adducts **meso-3** and **rac-3**. This transformation is suggested to involve thermal rearrangement of **1** to the intermediate 3,3'-dimethyl-1,1'-biisobenzofuran (**A**), and subsequent Diels-Alder cycloadditions of two equivalents of DMAD to **A**. The isolation of trapping products **meso-3** and **rac-3**, which contain complex polycyclic frameworks, provide strong evidence for the transient production of **A**, the first biisobenzofuran. An X-ray crystal structure of **meso-3** was obtained.

As part of a recent study of the thermal cyclization of η^2^-(*o*-ethynylbenzoyl)rhenium complexes to rhenium isobenzofuryl carbene complexes (Scheme 1),[1] we attempted to form a rhenium complex of the alkyne di-(*o*-acetylphenyl)acetylene (**1**) (Scheme 2). The addition of alkyne **1** to a solution of Cp(CO)_2_Re(THF) produced Cp(CO)_2_Re[η^2^-(di-(*o*-acetylphenyl)acetylene)] (**2**), which was detected by ^1^H NMR spectroscopy [δ 5.60 (Cp), 2.25 (Me)] (Scheme 2). However, we were unable to isolate **2**. In the absence of Cp(CO)_2_Re(THF), **1** underwent unexpected decomposition in dichloromethane (t_1/2_ ~ 20 h) to form an insoluble, uncharacterized yellow solid. The same yellow material was obtained both in the presence and absence of oxygen. This prompted further study into the nature of this unidentified decomposition process. Here we report evidence for a bicyclization reaction of **1** leading to the first biisobenzofuran, and its trapping by reaction with dimethyl acetylenedicarboxylate (DMAD). An X-ray crystal structure of the resulting bis Diels-Alder adduct of this highly unstable isobenzofuran is reported. Oligomeric isobenzofurans are theoretically interesting molecules and their optical and electronic properties have been studied computationally.[2–3]

**Scheme 1:**
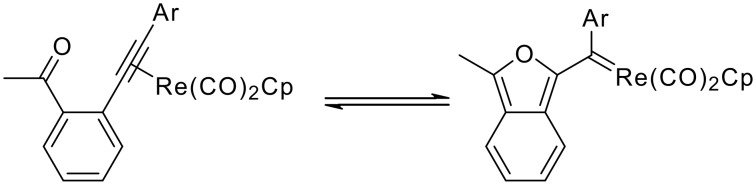
Thermal cyclization of η^2^-(*o*-ethynylbenzoyl)rhenium complexes to rhenium isobenzofuryl carbene complexes.

**Scheme 2:**
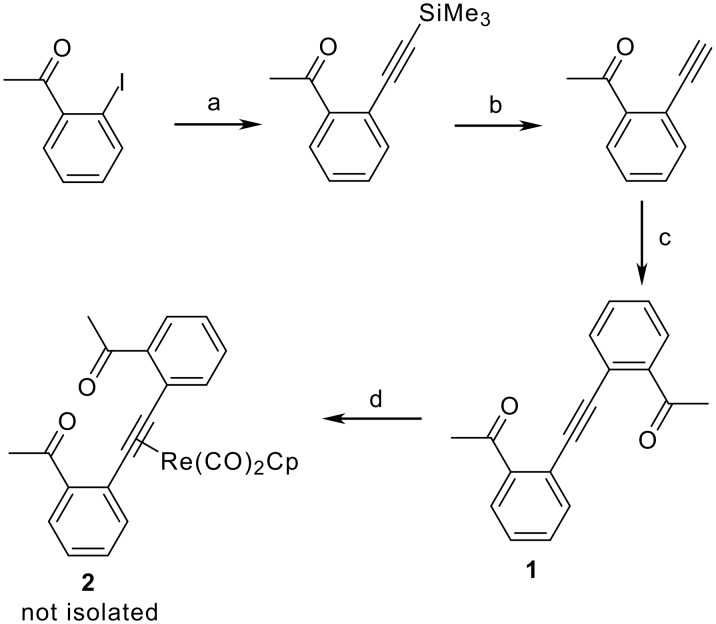
Reagents and conditions: (a) Me_3_SiC≡CH, Pd(PPh_3_)_4_, CuI, Et_3_N, toluene, 40°C, 22 h, 99 %; (b) KF, MeOH, 91 %; (c) 2'-iodoacetophenone, Pd(PPh_3_)_4_, CuI, Et_3_N, toluene, 40°C, 22 h, 62 %; (d) Cp(CO)_2_Re(THF), THF, 0→22°C, 5 h

Alkyne **1** was synthesized through two palladium catalyzed Sonogashira couplings (Scheme 2). Coupling of 2'-iodoacetophenone with trimethylsilylacetylene involving Pd(PPh_3_)_4_, CuI, and Et_3_N in toluene gave 2'-(trimethylsilylethynyl)acetophenone as a brown oil in 99% yield (See additional data file 1 for experimental details and spectral characterization of new compounds). Deprotection of 2'-(trimethylsilylethynyl)acetophenone was accomplished by reaction with anhydrous KF in MeOH and gave 2'-ethynylacetophenone in 91% yield. A second coupling of 2'-ethynylacetophenone with 2'-iodoacetophenone involving Pd(PPh_3_)_4_, CuI, and Et_3_N in toluene gave **1** as a yellow solid in 62% yield after recrystallization.

An NMR sample of **1** in CDCl_3_ left standing overnight showed significant decomposition leading to large amounts of insoluble yellow precipitate. We hypothesized that decomposition of **1** might involve rearrangement to an unseen intermediate 3,3'-dimethyl-1,1'-biisobenzofuran (**A**) and subsequent reaction with oxygen or dienophiles (Scheme 3). Isobenzofurans readily undergo reactions with oxygen, either in the presence or absence of light, to give diketones as the predominant products.[4–7] The bicyclization process considered for the conversion of **1** to **A** is analogous to the thermal rearrangement of a 2,6-alkadien-4-yn-1,8-dialdehyde to a bifuran observed by Iyoda[8] (Scheme 4) and to the related photoinduced bicyclization observed by Nakatani and Saito[9] (Scheme 5).

**Scheme 3:**
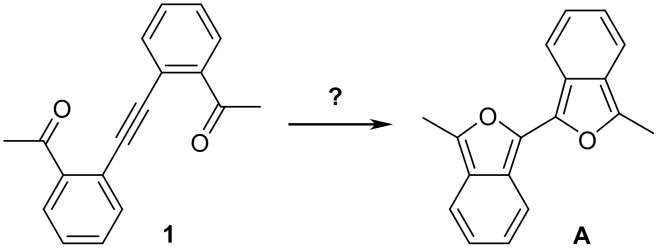
Possible thermal bicyclization of **1** to **A**.

**Scheme 4:**
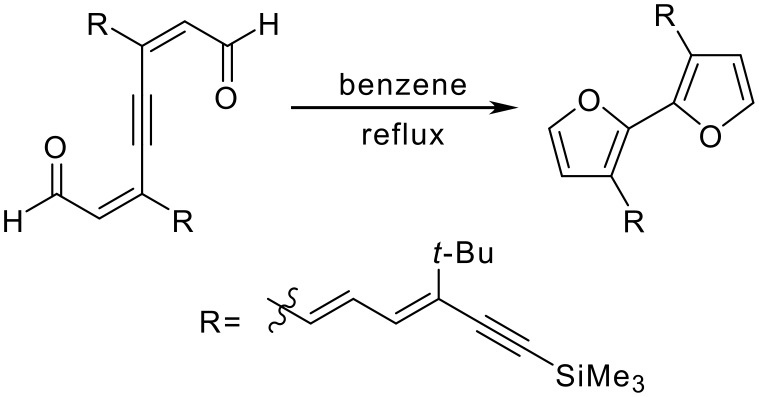
Thermal rearrangement of a 2,6-alkadien-4-yn-1,8-dialdehyde to a bifuran.

**Scheme 5:**
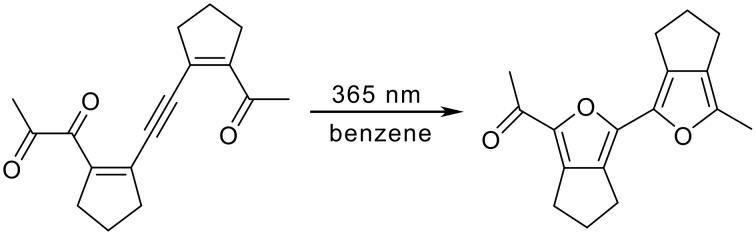
Photochemical cyclization to a bifuran.

Since suspected intermediate **A** proved too reactive to isolate, we sought to trap it with DMAD through Diels-Alder reactions with the isobenzofuran units. Acetylene **1** was dissolved in neat DMAD and the solution was degassed. After three days of stirring under N_2_, the excess DMAD was evaporated under vacuum (3 × 10^-2^ torr). Preparative TLC gave DMAD adducts **meso-3** and **rac-3** as white powders in 60% and 22% yields, respectively (Scheme 6). Both compounds were characterized spectroscopically through ^1^H and ^13^C NMR spectroscopy as well as by HRMS.

**Scheme 6:**
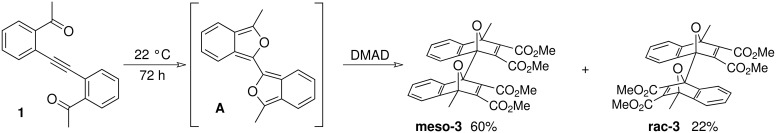
Trapping of **A** by DMAD to form Diels-Alder adducts **meso-3** and **rac-3**.

X-ray quality crystals of **meso-3** were obtained by slow diffusion of pentane into CH_2_Cl_2_ solution. In the X-ray crystal structure, **meso-3** adopts a *C_i_* symmetric conformation (Figure 1).

**Figure 1:**
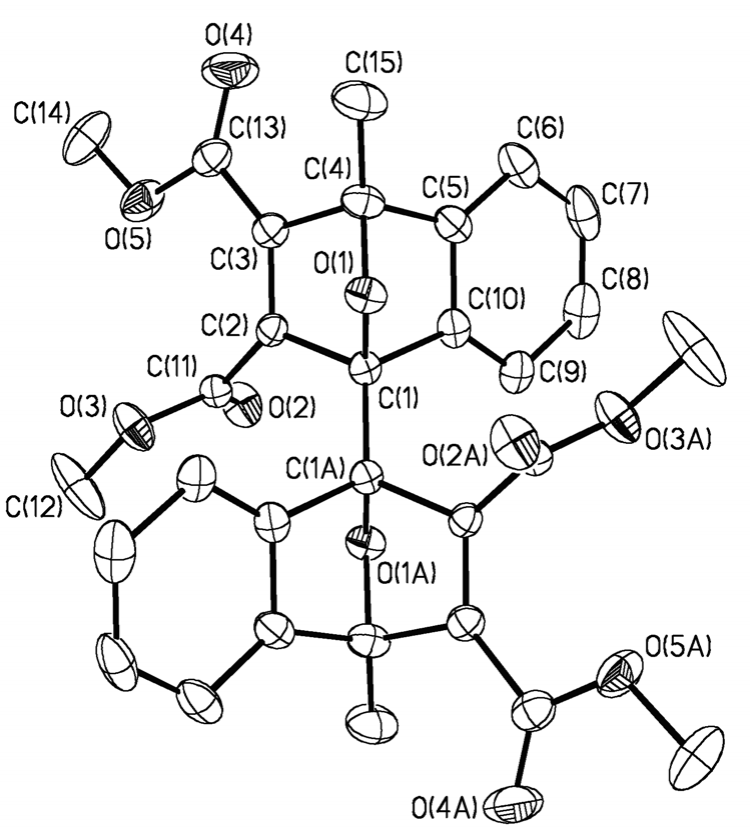
X-ray crystal structure of **meso-3**.

Isolation of DMAD adducts **meso-3** and **rac-3** provides strong evidence for the intermediacy of biisobenzofuran **A**. In the absence of a trapping agent, the formation of **A** is followed by rapid decomposition to the yellow uncharacterized solid. We can conceive of two pathways for the rearrangement of alkyne **1** to biisobenzofuran **A**. In the first, ring-forming nucleophilic attack of a carbonyl oxygen on the near carbon of the alkyne produces intermediate **B** (Scheme 7). This attack might be catalyzed by protonation of a carbonyl by adventitious acid. The enolate (or enol) oxygen of **B** would then attack the central carbon of the allene, closing the second ring and forming biisobenzofuran **A**.

**Scheme 7:**
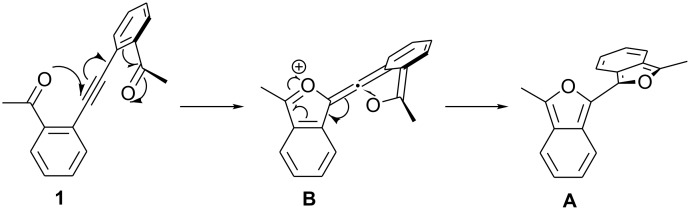
Possible stepwise mechanism for rearrangement of **1** to **A**.

In a second possible mechanism, **1** undergoes a concerted rearrangement where both rings are formed simultaneously through coupled electrocyclic processes. An analogy has been made between alkynes and 1,2-dicarbenes. Transformations in which strained alkynes formally react as dicarbenes have been observed.[10–14] Computations have supported the view that strained alkynes have dicarbene character.[15–16] The transformation of **1** to **A** can be viewed the alkyne reacting as a dicarbene (Scheme 8). Each carbene unit reacts as an analog of a 4-oxabutadienyl carbene, which are known to undergo 6π electrocyclic ring closures to furans.[9,17–18]

**Scheme 8:**
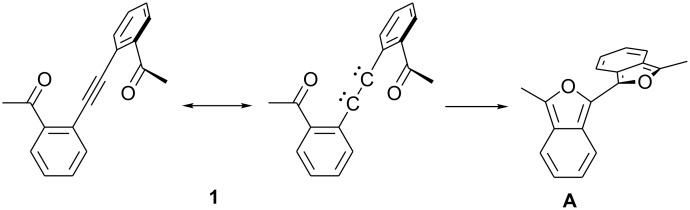
Possible concerted mechanism for rearrangement of **1** to **A**.

Related ring closures of *o*-acyl phenylcarbenes to isobenzofurans have been reported (Scheme 9).[19–21] These carbenes were formed as transient intermediates by photolytic or chemical cleavage of diazo or diazirine compounds. Isobenzofurans formed in this way were only detected in an argon matrix (~10 K) or through room temperature trapping experiments due to the inherent instability and high reactivity of isobenzofurans.

**Scheme 9:**
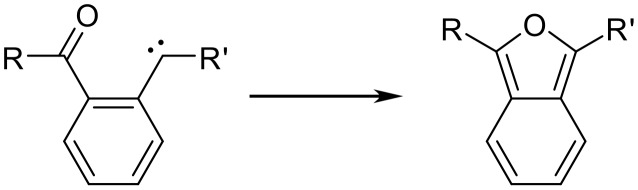
Ring closures of *o*-acyl phenylcarbenes to isobenzofurans.

The concerted bicyclization of **1** to **A** may also be related to the microscopic reverse of the thermal ring openings of 2-furylcarbenes to alk-2-en-4-yn-1-ones (Scheme 10). These ring openings have been studied both experimentally by matrix isolation spectroscopy [22–23] and computationally [24–25] and are described as involving coarctate transition states.[26] The transformation of **1** to **A** can be viewed as a coarctate ring closure coupled with a 6π electrocyclic ring closure of an *o*-acyl phenylcarbene (Scheme 11). This would require that the two rings be formed in planes perpendicular to one another since orthogonal orbitals of the alkyne are employed.

**Scheme 10:**
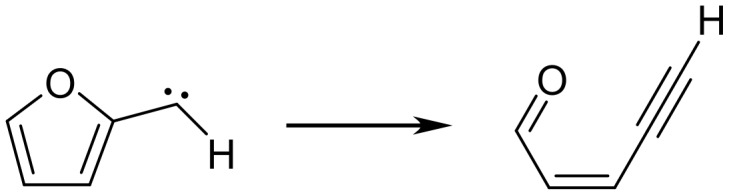
Ring opening of 2-furylcarbenes to alk-2-en-4-yn-1-ones.

**Scheme 11:**
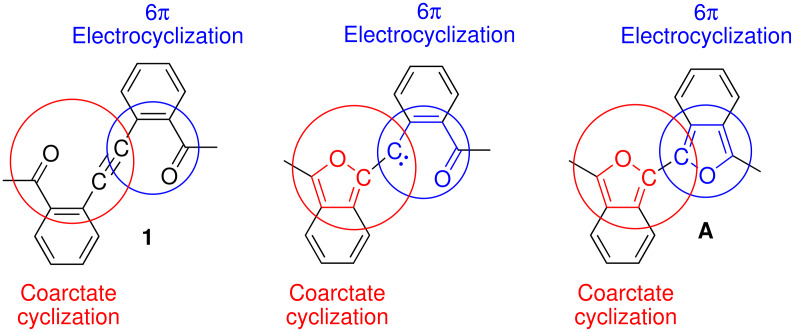
Coupled coarctate cyclization and 6π electrocyclization.

## Supporting Information Available

Experimental procedures and full spectroscopic data for all new compounds and X-ray crystallographic data for **meso-3** (16 pages). X-ray crystallographic data for **meso-3** has been deposited in the Cambridge Structural Database (CCDC # 289103).

## Supporting Information

File 1 bisfuran-supportinginfo.doc 100 Kb
