# Supplementary material for: Trapping evidence for the thermal cyclization of di-(o-acetylphenyl)acetylene to 3,3'-dimethyl-1,1'-biisobenzofuran
Source: Beilstein J Org Chem. 2005 Dec 9;1:18. doi: 10.1186/1860-5397-1-18 (PMC1399466; doi:10.1186/1860-5397-1-18)
Supplement: File 1 — bisfuran-supportinginfo.doc 100 Kb [file Beilstein_J_Org_Chem-01-18-s001.doc]

**Trapping Evidence for the Thermal Cyclization of**

**Di-(*o*-acetylphenyl)acetylene to 3,3’-Dimethyl-1,1’-biisobenzofuran**

Charles P. Casey,* Neil A. Strotman, and Ilia A. Guzei

*University of Wisconsin-Madison, Madison, WI 53706*

[casey@chem.wisc.edu](mailto:casey@chem.wisc.edu), strotman@chem.wisc.edu

**Additional Material**

**Experimental**

**2’-(Trimethylsilylethynyl)acetophenone.** Trimethylsilylacetylene (2.88 mL, 20.5 mmol) was added to a suspension of 2’-iodoacetophenone (2.32 mL, 16.3 mmol), Pd(PPh_3_)_4_ (378 mg, 0.326 mmol), CuI (155 mg, 0.816 mmol), Et_3_N (4.32 mL, 31.0 mmol), and toluene (120 mL) in a Schlenk flask. This suspension was heated to 40 °C and stirred under N_2_ for 24 h. Saturated NH_4_Cl_(aq)_ (110 mL) was added and the mixture was extracted with EtOAc (3 x 60 mL). The organic extract was dried (MgSO_4_), filtered, and concentrated under vacuum to give 2’-(trimethylsilylethynyl)acetophenone (3.50 g, 99%) as a brown oil. ^1^H NMR (300 MHz, CDCl_3_) δ 7.68 (d, *J* = 7.5 Hz, 1H), 7.56 (d, *J* = 7.5 Hz, 1H), 7.42 (td, *J* = 7.5, 1.7 Hz, 1H), 7.37 (td, *J* = 7.5, 1.7 Hz, 1H), 2.75 (s, 3H), 0.26 (s, 9H). ^13^C {^1^H} NMR (75.4 MHz, CDCl_3_) δ 200.9, 141.7, 134.4, 131.3, 128.8, 128.6, 121.6, 104.0, 101.3, 30.3, -0.1 (3C). HRMS (ESI) calcd for C_13_H_16_OSiNa (M+Na)^+^, 239.0868; found, 239.0874.

**2’-Ethynylacetophenone.** 2’-(Trimethylsilylethynyl)acetophenone (3.50 g, 16.2 mmol) was added to a suspension of anhydrous KF (6.59 g, 0.113 mol) in MeOH (145 mL). After stirring under N_2_ for 2 h, ~2/3 of the MeOH was evaporated by rotary evaporation and Et_2_O (700 mL) and H_2_O (250 mL) were added. The organic layer was separated, washed with H_2_O (2 x 250 mL), dried (MgSO_4_), filtered, and concentrated by rotary evaporation to give 2’-ethynylacetophenone as a pale brown oil (2.13 g, 91%). ^1^H NMR (CDCl_3_, 300 MHz) δ 7. 71 (d, *J* = 6.8 Hz, 1H), 7.60 (d, *J* = 6.6 Hz, 1H), 7.45 (td, *J* = 7.5, 1.8 Hz, 1H), 7.41 (td, *J* = 7.5, 1.8 Hz, 1H), 3.40 (s, 1H), 2.72 (s, 3H). ^13^C {^1^H} NMR (75.4 MHz, CDCl_3_) δ 200.3, 141.7, 134.9, 131.4, 129.0, 128.7, 120.6, 83.1, 82.6, 30.0. MS (EI) [m/z (% abundance)] 145 (4), 144 (35), 129 (60), 101 (35), 88 (39), 86 (98), 85 (28), 84 (100), 83 (37). HRMS (EI) calcd for C_10_H_8_O (M^+^), 144.0575; found, 144.0574.

**Di-(o-acetylphenyl)acetylene (1)**. 2’-Ethynylacetophenone (0.65 g, 4.5 mmol) was added to a suspension of 2’-iodoacetophenone (0.64 mL, 4.5 mmol), Pd(PPh_3_)_4_ (104 mg, 0.90 mmol), CuI (43 mg, 0.23 mmol), Et_3_N (1.19 mL, 8.6 mmol), and toluene (30 mL) in a Schlenk flask. This suspension was heated to 40 °C and stirred under N_2_ for 6 h. Saturated NH_4_Cl_(aq)_ (30 mL) was added and the mixture was extracted with EtOAc (3 x 20 mL). Evaporation of solvents from the organic layer gave an oily orange solid, which was redissolved in Et_2_O (10 mL). Overnight at –30 °C, di-(o-acetylphenyl)acetylene crystallized as fluffy yellow crystals (0.73 g, 62%). ^1^H NMR (300 MHz, CD_2_Cl_2_) δ 7.77 (ddd, *J* = 7.8, 1.2, 0.6 Hz, 2H), 7.68 (ddd, *J* = 7.5, 1.5, 0.6 Hz, 2H), 7.53 (td, *J* = 7.8, 1.5 Hz, 2H), 7.45 (td, *J* = 7.5, 1.5 Hz, 1H), 2.74 (s, 6H). ^13^C {^1^H} NMR (75.4 MHz, CD_2_Cl_2_) δ 200.0, 141.1, 134.5, 131.9, 129.3, 129.2, 121.9, 94.0, 30.1. IR(CH_2_Cl_2_): 1688 (s) cm^-1^. HRMS (ESI) calcd for C_18_H_14_O_2_Na (M+Na)^+^, 285.0891; found, 285.0891.

**Dimethylacetylene Dicarboxylate Diels-Alder Adducts Meso-3 and Rac-3**. Di-(*o*-acetylphenyl)acetylene (34.9 mg, 0.133 mmol) was combined with dimethylacetylenedicarboxylate (DMAD) (1.80 mL, 14.6 mmol) and the solution was degassed under vacuum. After 3 days of stirring at room temperature in the dark under N_2_, the DMAD was evaporated under vacuum (3 x 10^-2^ torr). The resulting orange solid was purified by preparative TLC (silica gel, 1:5 pentane:ether) to give **meso-3** (R_f_ = 0.60) as a white powder (43.6 mg, 60%) and **rac-3** (R_f_ = 0.47) as an off-white powder (15.6 mg, 22%). X-ray quality crystals of **meso-3** were obtained by slow diffusion of pentane into a CH_2_Cl_2_ solution. For **meso-3**: ^1^H NMR (300 MHz, CD_2_Cl_2_) δ 7.62 (d, *J* = 7.4 Hz, 2H), 7.35 (d, *J* = 7.4 Hz, 2H), 7.15 (td, *J* = 7.4, 1.5 Hz, 2H), 7.11 (td, *J* = 7.4, 1.5 Hz, 2H), 3.67 (s, 6H), 3.38 (s, 6H), 2.13 (s, 6H). ^13^C {^1^H} NMR (126 MHz, CD_2_Cl_2_) δ 164.7, 162.7, 157.3, 149.7, 147.8, 147.6, 126.9, 126.3, 123.4, 120.0, 92.0, 90.5, 52.6, 52.4, 15.0. HRMS (ESI) calcd for C_30_H_26_O_10_Na (M+Na)^+^, 569.1424; found, 569.1435. For **rac-3**, ^1^H NMR (300 MHz, CD_2_Cl_2_) δ 7.35 (d, *J* = 7.7Hz, 2H), 7.08 (td, *J* = 7.7, 1.1 Hz, 2H), 7.07 (d, *J* = 7.7 Hz, 2H), 6.91 (td, *J* = 7.7, 1.1 Hz, 2H), 3.71 (s, 6H), 3.69 (s, 6H), 2.11 (s, 6H). ^13^C {^1^H} NMR (126 MHz, CD_2_Cl_2_) δ 164.8, 163.0, 156.7, 150.4, 147.6, 147.5, 126.7, 126.0, 123.3, 120.2, 92.0, 90.6, 52.7, 52.6, 14.8. IR(CH_2_Cl_2_): 1740 (s), 1717 (s), 1632 (m) cm^-1^. HRMS (ESI) calcd for C_30_H_26_O_10_Na (M+Na)^+^, 569.1424; found, 569.1439.

**X-Ray Crystal Structure Determination of meso-3**

## Data Collection

A colorless crystal with approximate dimensions 0.38 x 0.31 x 0.11 mm^3^ was selected under oil under ambient conditions and attached to the tip of a glass fiber at RT.

The crystal evaluation and data collection were performed on a Bruker CCD-1000 diffractometer with Mo K_α_ (λ = 0.71073 Å) radiation and the diffractometer to crystal distance of 4.9 cm.

The initial cell constants were obtained from three series of ω scans at different starting angles. Each series consisted of 20 frames collected at intervals of 0.3º in a 6º range about ω with the exposure time of 30 seconds per frame. A total of 34 reflections was obtained. The reflections were successfully indexed by an automated indexing routine built in the SMART program. The final cell constants were calculated from a set of 2181 strong reflections from the actual data collection.

The data were collected by using the hemisphere data collection routine. The reciprocal space was surveyed to the extent of a full sphere to a resolution of 0.80 Å. A total of 5100 data were harvested by collecting three sets of frames with 0.3º scans in ω with an exposure time 90 sec per frame. These highly redundant datasets were corrected for Lorentz and polarization effects. The absorption correction was based on fitting a function to the empirical transmission surface as sampled by multiple equivalent measurements. [1]

### Structure Solution and Refinement

The systematic absences in the diffraction data were consistent for the space groups *P*1 and *P*1. The *E*-statistics strongly suggested the centrosymmetric space group *P*1 that yielded chemically reasonable and computationally stable results of refinement [1]. A successful solution by the direct methods provided most non-hydrogen atoms from the *E*-map. The remaining non-hydrogen atoms were located in an alternating series of least-squares cycles and difference Fourier maps. All non-hydrogen atoms were refined with anisotropic displacement coefficients. All hydrogen atoms were included in the structure factor calculation at idealized positions and were allowed to ride on the neighboring atoms with relative isotropic displacement coefficients. There are two symmetry independent half-molecules in the asymmetric unit. Each molecules occupies a crystallographic inversion center.

The final least-squares refinement of 367 parameters against 3671 data resulted in residuals *R* (based on *F*^2^ for *I*≥2*σ*) and *wR* (based on *F*^2^ for all data) of 0.0652 and 0.2065, respectively. The final difference Fourier map was featureless.

The ORTEP diagrams are drawn with 30% probability ellipsoids.

### References

[1] Bruker-AXS. (2000-2003) SADABS V.2.05, SAINT V.6.22, SHELXTL V.6.10

& SMART 5.622 Software Reference Manuals. Bruker-AXS, Madison, Wisconsin, USA.

Figure 1. The first symmetry independent molecule.

Figure 2. The second symmetry independent molecule.

Figure 3. The two unique parts of each molecule superimposed.

Table 1. Crystal data and structure refinement for **meso-3**.

Empirical formula C_30_ H_26_ O_10_

Formula weight 546.51

Temperature 296(2) K

Wavelength 0.71073 Å

Crystal system Triclinic

Space group P1

Unit cell dimensions a = 9.4041(11) Å α= 91.815(4)°.

b = 10.2960(12) Å β= 100.474(4)°.

c = 14.5881(17) Å γ = 98.107(4)°.

Volume 1372.7(3) Å^3^

Z 2

Density (calculated) 1.322 Mg/m^3^

Absorption coefficient 0.100 mm^-1^

F(000) 572

Crystal size 0.38 x 0.31 x 0.11 mm^3^

Theta range for data collection 2.00 to 23.50°.

Index ranges -10<=h<=6, -9<=k<=11, -10<=l<=16

Reflections collected 5100

Independent reflections 3671 [R(int) = 0.0157]

Completeness to theta = 23.50° 90.1 %

Absorption correction Multiscan with SADABS

Max. and min. transmission 0.9891 and 0.9630

Refinement method Full-matrix least-squares on F^2^

Data / restraints / parameters 3671 / 0 / 367

Goodness-of-fit on F^2^ 0.967

Final R indices [I>2sigma(I)] R1 = 0.0652, wR2 = 0.1942

R indices (all data) R1 = 0.1014, wR2 = 0.2065

Largest diff. peak and hole 0.285 and -0.286 e.Å^-3^

Table 2. Atomic coordinates ( x 10^4^) and equivalent isotropic displacement parameters (Å^2^x 10^3^)

for **meso-3**. U(eq) is defined as one third of the trace of the orthogonalized Uij tensor.

________________________________________________________________________________

x y z U(eq)

________________________________________________________________________________

O(1) 4478(3) 607(3) 8839(2) 39(1)

O(2) 3885(4) 1932(4) 11730(2) 53(1)

O(3) 5982(4) 2861(4) 11378(2) 54(1)

O(4) 1974(6) 3777(5) 8853(3) 87(2)

O(5) 3381(5) 4218(3) 10263(3) 59(1)

O(6) -155(3) 4555(3) 3750(2) 36(1)

O(7) 2571(5) 7995(4) 5626(3) 71(1)

O(8) 230(4) 8156(4) 5186(3) 62(1)

O(9) 2976(5) 7068(4) 2538(3) 76(1)

O(10) 2256(5) 8576(4) 3407(3) 68(1)

C(1) 4379(5) 333(4) 9783(3) 31(1)

C(2) 4264(5) 1722(4) 10174(3) 32(1)

C(3) 3525(5) 2277(5) 9466(3) 37(1)

C(4) 3182(6) 1241(5) 8624(3) 44(1)

C(5) 2046(5) 177(5) 8892(4) 42(1)

C(6) 573(6) -210(6) 8578(4) 56(2)

C(7) -125(6) -1208(7) 9030(5) 66(2)

C(8) 620(7) -1765(6) 9775(5) 64(2)

C(9) 2122(6) -1359(5) 10113(4) 44(1)

C(10) 2797(5) -403(5) 9648(4) 37(1)

C(11) 4663(5) 2173(5) 11180(3) 33(1)

C(12) 6502(8) 3326(9) 12343(4) 105(3)

C(13) 2880(6) 3500(5) 9474(4) 47(1)

C(14) 2785(8) 5429(6) 10349(5) 75(2)

C(15) 2985(7) 1706(6) 7650(4) 66(2)

C(16) 536(5) 5090(4) 4674(3) 32(1)

C(17) 1095(5) 6506(5) 4436(3) 36(1)

C(18) 1451(5) 6379(5) 3604(3) 37(1)

C(19) 1108(6) 4891(5) 3303(3) 40(1)

C(20) 2247(5) 4247(5) 3958(3) 38(1)

C(21) 3384(6) 3642(5) 3829(4) 51(2)

C(22) 4223(7) 3175(6) 4611(5) 67(2)

C(23) 3889(6) 3351(6) 5482(5) 58(2)

C(24) 2729(6) 3972(5) 5619(4) 44(1)

C(25) 1890(5) 4413(5) 4842(3) 37(1)

C(26) 1417(6) 7650(5) 5139(4) 39(1)

C(27) 339(10) 9085(7) 5975(5) 100(3)

C(28) 2285(6) 7354(6) 3108(4) 48(1)

C(29) 3181(9) 9596(6) 3037(6) 89(2)

C(30) 749(7) 4448(6) 2285(4) 58(2)

________________________________________________________________________________

Table 3. Bond lengths [Å] and angles [°] for **meso-3**.

_____________________________________________________

O(1)-C(1) 1.432(5)

O(1)-C(4) 1.452(6)

O(2)-C(11) 1.190(5)

O(3)-C(11) 1.316(6)

O(3)-C(12) 1.445(7)

O(4)-C(13) 1.196(6)

O(5)-C(13) 1.320(7)

O(5)-C(14) 1.449(7)

O(6)-C(16) 1.441(5)

O(6)-C(19) 1.461(6)

O(7)-C(26) 1.184(6)

O(8)-C(26) 1.308(6)

O(8)-C(27) 1.453(7)

O(9)-C(28) 1.197(6)

O(10)-C(28) 1.325(7)

O(10)-C(29) 1.448(7)

C(1)-C(1)#1 1.489(9)

C(1)-C(10) 1.547(6)

C(1)-C(2) 1.549(6)

C(2)-C(3) 1.329(6)

C(2)-C(11) 1.489(7)

C(3)-C(13) 1.473(7)

C(3)-C(4) 1.557(7)

C(4)-C(15) 1.502(7)

C(4)-C(5) 1.530(7)

C(5)-C(6) 1.375(7)

C(5)-C(10) 1.395(7)

C(6)-C(7) 1.389(9)

C(7)-C(8) 1.370(9)

C(8)-C(9) 1.407(8)

C(9)-C(10) 1.362(7)

C(16)-C(16)#2 1.504(9)

C(16)-C(25) 1.522(7)

C(16)-C(17) 1.551(6)

C(17)-C(18) 1.324(7)

C(17)-C(26) 1.497(7)

C(18)-C(28) 1.475(7)

C(18)-C(19) 1.553(7)

C(19)-C(30) 1.504(7)

C(19)-C(20) 1.538(7)

C(20)-C(21) 1.348(7)

C(20)-C(25) 1.402(7)

C(21)-C(22) 1.403(8)

C(22)-C(23) 1.376(9)

C(23)-C(24) 1.381(8)

C(24)-C(25) 1.387(7)

C(1)-O(1)-C(4) 97.6(3)

C(11)-O(3)-C(12) 116.3(4)

C(13)-O(5)-C(14) 115.9(5)

C(16)-O(6)-C(19) 97.0(3)

C(26)-O(8)-C(27) 115.5(5)

C(28)-O(10)-C(29) 116.2(5)

O(1)-C(1)-C(1)#1 110.6(5)

O(1)-C(1)-C(10) 100.7(4)

C(1)#1-C(1)-C(10) 119.6(5)

O(1)-C(1)-C(2) 100.0(3)

C(1)#1-C(1)-C(2) 119.0(5)

C(10)-C(1)-C(2) 103.8(4)

C(3)-C(2)-C(11) 129.3(4)

C(3)-C(2)-C(1) 105.2(4)

C(11)-C(2)-C(1) 124.5(4)

C(2)-C(3)-C(13) 128.9(5)

C(2)-C(3)-C(4) 106.2(4)

C(13)-C(3)-C(4) 124.0(4)

O(1)-C(4)-C(15) 111.3(5)

O(1)-C(4)-C(5) 99.9(4)

C(15)-C(4)-C(5) 120.2(5)

O(1)-C(4)-C(3) 98.9(4)

C(15)-C(4)-C(3) 119.0(5)

C(5)-C(4)-C(3) 104.0(4)

C(6)-C(5)-C(10) 120.7(5)

C(6)-C(5)-C(4) 134.0(5)

C(10)-C(5)-C(4) 105.2(4)

C(5)-C(6)-C(7) 117.5(6)

C(8)-C(7)-C(6) 121.3(6)

C(7)-C(8)-C(9) 121.6(6)

C(10)-C(9)-C(8) 116.3(6)

C(9)-C(10)-C(5) 122.5(5)

C(9)-C(10)-C(1) 133.6(5)

C(5)-C(10)-C(1) 103.9(4)

O(2)-C(11)-O(3) 124.8(5)

O(2)-C(11)-C(2) 123.9(4)

O(3)-C(11)-C(2) 111.3(4)

O(4)-C(13)-O(5) 124.6(5)

O(4)-C(13)-C(3) 123.7(5)

O(5)-C(13)-C(3) 111.7(5)

O(6)-C(16)-C(16)#2 110.2(5)

O(6)-C(16)-C(25) 101.2(3)

C(16)#2-C(16)-C(25) 119.9(5)

O(6)-C(16)-C(17) 99.2(3)

C(16)#2-C(16)-C(17) 118.4(5)

C(25)-C(16)-C(17) 104.8(4)

C(18)-C(17)-C(26) 130.7(4)

C(18)-C(17)-C(16) 105.2(4)

C(26)-C(17)-C(16) 122.9(4)

C(17)-C(18)-C(28) 129.0(5)

C(17)-C(18)-C(19) 106.6(4)

C(28)-C(18)-C(19) 123.3(5)

O(6)-C(19)-C(30) 111.1(4)

O(6)-C(19)-C(20) 99.7(4)

C(30)-C(19)-C(20) 118.7(5)

O(6)-C(19)-C(18) 98.2(4)

C(30)-C(19)-C(18) 120.1(4)

C(20)-C(19)-C(18) 105.3(4)

C(21)-C(20)-C(25) 121.8(5)

C(21)-C(20)-C(19) 133.9(5)

C(25)-C(20)-C(19) 104.2(4)

C(20)-C(21)-C(22) 118.3(5)

C(23)-C(22)-C(21) 120.1(5)

C(22)-C(23)-C(24) 122.0(6)

C(23)-C(24)-C(25) 117.7(5)

C(24)-C(25)-C(20) 120.1(5)

C(24)-C(25)-C(16) 134.9(4)

C(20)-C(25)-C(16) 105.0(4)

O(7)-C(26)-O(8) 124.7(5)

O(7)-C(26)-C(17) 124.3(5)

O(8)-C(26)-C(17) 110.7(5)

O(9)-C(28)-O(10) 123.9(5)

O(9)-C(28)-C(18) 123.7(5)

O(10)-C(28)-C(18) 112.3(5)

_____________________________________________________________

Symmetry transformations used to generate equivalent atoms:

#1 -x+1,-y,-z+2 #2 -x,-y+1,-z+1

Table 4. Anisotropic displacement parameters (Å^2^x 10^3^) for **meso-3**. The anisotropic

displacement factor exponent takes the form: -2π2[ h^2^ a*^2^U^11^ + ... + 2 h k a* b* U^12^ ]

______________________________________________________________________________

U^11^ U^22^ U^33^ U^23^ U^13^ U^12^

______________________________________________________________________________

O(1) 35(2) 48(2) 36(2) 1(2) 10(2) 13(2)

O(2) 45(2) 70(3) 45(2) -1(2) 23(2) -3(2)

O(3) 36(2) 82(3) 39(2) -13(2) 11(2) -8(2)

O(4) 101(4) 78(3) 79(3) 6(3) -19(3) 50(3)

O(5) 83(3) 41(2) 59(3) 0(2) 17(2) 25(2)

O(6) 35(2) 47(2) 30(2) -2(2) 14(2) 10(2)

O(7) 52(3) 82(3) 70(3) -23(2) -5(2) 9(2)

O(8) 61(3) 53(2) 76(3) -14(2) 18(2) 20(2)

O(9) 94(3) 68(3) 81(3) 6(2) 63(3) 7(3)

O(10) 88(3) 48(2) 79(3) 10(2) 47(3) 5(2)

C(1) 31(3) 32(3) 32(3) -1(2) 8(2) 5(2)

C(2) 31(3) 30(3) 37(3) 3(2) 8(2) 5(2)

C(3) 35(3) 39(3) 40(3) 2(2) 10(2) 9(2)

C(4) 40(3) 54(3) 38(3) 3(3) 5(2) 17(3)

C(5) 36(3) 47(3) 43(3) -11(3) 2(3) 15(3)

C(6) 31(3) 67(4) 68(4) -25(3) -1(3) 18(3)

C(7) 28(3) 65(4) 100(5) -41(4) 7(4) 4(3)

C(8) 44(4) 50(4) 103(5) -18(4) 36(4) 0(3)

C(9) 37(3) 37(3) 62(4) -7(3) 21(3) 9(3)

C(10) 26(3) 38(3) 50(3) -8(2) 13(2) 8(2)

C(11) 27(3) 36(3) 37(3) 0(2) 9(2) 6(2)

C(12) 61(4) 182(9) 46(4) -36(5) 0(3) -41(5)

C(13) 46(3) 45(3) 53(4) 11(3) 15(3) 9(3)

C(14) 113(6) 43(3) 89(5) 13(3) 58(4) 27(4)

C(15) 76(4) 84(5) 38(3) 5(3) -1(3) 24(4)

C(16) 28(3) 38(3) 35(3) -1(2) 13(2) 15(2)

C(17) 36(3) 34(3) 42(3) 4(2) 14(2) 13(2)

C(18) 35(3) 46(3) 37(3) 8(2) 18(2) 14(2)

C(19) 41(3) 49(3) 36(3) -3(2) 20(2) 11(3)

C(20) 35(3) 39(3) 46(3) -1(2) 22(3) 8(3)

C(21) 40(3) 59(4) 68(4) 4(3) 38(3) 23(3)

C(22) 43(4) 74(4) 99(5) 12(4) 31(4) 32(3)

C(23) 43(4) 68(4) 71(4) 14(3) 16(3) 24(3)

C(24) 44(3) 50(3) 41(3) 6(3) 12(3) 13(3)

C(25) 36(3) 36(3) 40(3) 2(2) 15(2) 7(2)

C(26) 41(3) 35(3) 48(3) 4(2) 20(3) 15(3)

C(27) 133(7) 79(5) 96(6) -38(4) 34(5) 43(5)

C(28) 46(3) 52(4) 52(4) 7(3) 24(3) 14(3)

C(29) 110(6) 55(4) 114(6) 27(4) 59(5) -3(4)

C(30) 73(4) 68(4) 38(3) -1(3) 24(3) 9(3)

______________________________________________________________________________

Table 5. Hydrogen coordinates ( x 10^4^) and isotropic displacement parameters (Å^2^x 10 ^3^)

for **meso-3**.

________________________________________________________________________________

x y z U(eq)

________________________________________________________________________________

H(6) 62 182 8081 67

H(7) -1116 -1503 8822 79

H(8) 121 -2428 10064 77

H(9) 2626 -1724 10626 52

H(12A) 5916 3955 12509 157

H(12B) 7503 3734 12423 157

H(12C) 6432 2599 12736 157

H(14A) 2964 5966 9844 113

H(14B) 3244 5893 10932 113

H(14C) 1750 5231 10328 113

H(15A) 3797 2361 7599 99

H(15B) 2094 2077 7515 99

H(15C) 2939 977 7213 99

H(21) 3605 3537 3238 61

H(22) 5006 2746 4540 81

H(23) 4462 3043 5994 70

H(24) 2517 4089 6212 53

H(27A) 593 8661 6546 149

H(27B) -584 9390 5957 149

H(27C) 1079 9818 5944 149

H(29A) 4175 9632 3355 134

H(29B) 2862 10428 3130 134

H(29C) 3119 9403 2382 134

H(30A) 326 3538 2216 87

H(30B) 1626 4562 2029 87

H(30C) 64 4963 1959 87

________________________________________________________________________________
